# Medical Society of the State of New York—(Concluded)

**Published:** 1868-03

**Authors:** 


### Miscellaneous.

### Medical Society of the State of New York—(Concluded.)

### Sixty-First Anniversary.

THIRD DAY—MORNING SESSION.

The meeting was called to order by the President, and after a prayer by the Rev. Dr. Reese of Albany, the minutes of the previous meeting were read and approved.

*Delegates to International Medical Congress.*—Dr. Brinsmade, on behalf of the Committee of Delegates to the International Medical Congress at Paris, made the following report:

The undersigned, delegates from this Society to the "International Medical Congress," held at Paris on the 16th day of August, 1867, respectfully report, that they might make sure of being duly admitted to the Congress, they called on the 15th of August, at the office of the Secretary, M. Jaccoud, No. 4, Rue Brouot, with the certificates of their appointment by this Society, and after sending up cards and waiting for some time in the ante-room, we were informed by the female concièrge that M. Jaccoud was engaged, but that we might record our names in a little book lying upon the table, and take a green "carte de membre adhérent," on which were the names of the President, M. Bouillaud, and the Secretary, M. Jaccoud, the filling up of which we were allowed to do as we might think proper.

Inasmuch as our credentials were not required we of course reserved them for future use, if necessary. They were *never* called for, or examined by any officer or committee, showing to us that less discrimination was observed in order to give character and distinction to a scientific assembly than we were accustomed to in our own country. The card informed us of the *day* of the first séance, but neither the place nor hour of meeting was designated. After not a little inquiry we learned that the place was the great Hall of the "Faculty of Medicine." On presenting ourselves at the door, no cards or certificates were demanded, and we entered with the crowd to find seats for ourselves. The bare benches of the amphitheatre were arranged in the usual semicircular manner, without any support for the back; and gentlemen were compelled to step upon them to reach the forward seats, and in sitting were forced to use the one in front for their feet, bringing boots and coat-skirts in disagreeable proximity. The room was indeed a large one, but so imperfectly ventilated that respiration was quite difficult after remaining a few minutes. The dirty and uncomfortable seats, the heat, and the irrespirable atmosphere, prevented a majority of the audience from remaining during the entire session.

It did seem to us that in the elegant city of Paris pleasanter accommodations might have been secured wherein to receive *invited* guests, who were the accredited representatives of governments and of learned societies from all parts of the world. Your delegates deemed themselves honored by their appointment to represent so large a Society from so large a State as that of New York, equaling in population and size several of the kingdoms of Europe; and confess that they felt themselves, in virtue of their office, entitled to some consideration.

But, according to French arrangements, *any* person having access to the ante-room of M. Jaccoud could avail himself of all the privileges procurable by the green ticket.

About the 6th of January last your delegates received through the mail a ticket which seems to be intended as an acknowledgment — perhaps rather tardy — that they were entitled to the "Carte de membre adhérent." A large proportion of the audience seemed to be young men, perhaps young physicians, and medical students of Paris and vicinity. There were present, however, adding grace and dignity to the assembly, from cities, towns, and schools of continental Europe, many of the most distinguished medical men of the world.

Great Britain and Ireland sent but few, scarcely any, of their great surgeons and physicians. America was represented by some of our ablest men.

The names of Virchow of Berlin, Halla of Prague, De Méric of Brussels, Bérard of Montpelier, Baron Larry and Ricord of Paris, Frérichs of Berlin, Ernest Hart of London, Sangalli of Pavia, and of many others of equal celebrity, would confer honor upon any congress.

It was announced by the Secretary that on Saturday evening, August 24th, a banquet would be prepared at "Le Grande Hotel" for those who would send their names, with a Napoleon, to the

Secretary. About two hundred gentlemen were present at the dinner—members of the Congress, and other distinguished physicians from Paris and other cities on the continent. The dinner was as at "Tables des Hôtes" in first-class hotels, with perhaps a few more courses, and a greater variety of fine wines. After dinner brief speeches were made by the President, M. Bouillaud, M. Palasciano of Naples, M. Teissier of Lyons, M. Jaccoud, M. Galligo of Florence, M. Piorry, M. Ricord, and Mr. Ernest Hart of London.

Your delegates received the most polite attentions from many distinguished members of the profession in Paris, and, although they feel that the result of the meeting has not been as useful to the cause of medical science as was anticipated, nor the extension of acquaintance equal to what was hoped for, yet many pleasant associations were formed, a healthful, vigorous, and generous emulation was excited, a wholesome and dignified "esprit de corps" was experienced; and I doubt not most of us, when recalling to ourselves the many pleasant and intelligent faces we have seen, the cordial greetings we have received, the evidence of ability and zeal manifested by medical brethren from various countries, will forget the uncomfortable seats, the irrespirability of the atmosphere of the hall, and will be willing to make liberal allowance for differences in the arrangements and manner of conducting scientific associations and social reunions in different countries, and will remember with gratitude the originators and managers of the International Medical Congress, and this Society, who conferred the honor and gave us the privilege of representing it in this first attempt at an international assembly of men who do honor to their race by their benevolent efforts, and with whom all may justly feel pride in associating. The subjects of the papers which were read and discussed were some of the most important in medical science, and their character can be judged from the abstracts which have been published, and from the great ability of the authors and speakers. There was much talent exhibited, and many facts, deductions, principles and practices were brought out which will make the first International Medical Congress long memorable.

It would be useless for your delegates to attempt anything like an analysis of the papers, as they will doubtless be published separately or together; and such ample notices of them have appeared in various medical journals, that members can obtain more correct views of their value than we shall be able to present in this report; and we simply subjoin the programme, giving the subjects in the order of their consideration, thinking it may be useful as a matter of reference.

THOMAS C. BRINSMADE,
JOSEPH C. HUTCHISON,
HENRY S. DOWNS.

*Registration of Births and Marriages in the State.*—Dr. Lansing, as chairman of the committee to present to the Legislature the subject of registration of births and marriages in this State, etc., made the following report, which was adopted:

The committee appointed at the last meeting of this Society, to present to the Legislature the subject of the registration of births, deaths, and marriages in this State, and to solicit the passage of a law providing for a thorough and efficient system of such registration, respectfully report:

That a hearing was granted your committee by the Judiciary Committee, both of the Senate and Assembly of 1866–67, and the subject duly brought before them, with such explanations and arguments on the part of your committee as seemed suitable to the occasion. The result of such interviews was an unfavorable report on the part of the committee of the Senate, who, while conceding the importance and desirability of such a law, seemed to doubt if it could be practically carried out, and to stumble at the fact of the present existing law on the subject, which remains a dead letter on the statute book.

The committee of the Assembly noticed the subject favorably, and reported a bill substantially the same as the one contained in our last volume of Transactions, and the same was printed and placed on the files of the Assembly.

Owing to the multiplicity of other business before the Assembly, and the late period of the session at which it was brought before them, the matter failed of further progress.

The next step of your committee was to bring the subject before the Governor, and a suitable memorial was prepared and submitted to him, with the expectation that he would communicate the same to the Legislature at its present session, with favorable recommendations for the passage of some such law as is desired by this Society.

It is believed that in such expectation your committee will not be disappointed. Such is the present position of the matter in charge of your committee. Its final success in the Legislature would seem to depend upon the merits of the measure itself, and the exertions of its friends.

Respectfully submitted,

T. F. BRINSMADE,
JOHN V. LANSING,
J. S. MOSHER,
F. B. HOUGH.

The report of the Nominating Committee was received, and on motion, it was adopted.

It was then moved to substitute the name of John P. Garrish for permanent member in the first district, for that of J. Marion Sims. Adopted.

The President, according to the action of the Society, cast a ballot in favor of the officers named by the Nominating Committee, excepting the first district, for which the Society balloted separately, with the result of the election of W. B. Bibbins and S. D. Hubbard.

*Officers Elect for* 1868.—The following is the report of the Committee of Nominations:

For President—Dr. J. V. P. Quackenbush, Albany.
" Vice President—Dr. James P. White, Buffalo.
" Secretary—William H. Bailey, Albany.
" Treasurer—J. V. Lansing, Albany.

For Censors, Southern District—J. F. Jenkins, of Yonkers; Samuel H. Purdy, of New York; Edward R. Squibb, of Brooklyn. Eastern District—B. P. Staats, of Albany; T. C. Brinsmade, of Troy; P. McNaughton, of Albany. Middle District—M. M. Bagg, of Utica; C. B. Coventry, of Utica; A. F. Doolittle, of Herkimer. Western District—Sandford Eastman, of Buffalo; Edward Hall, of Auburn; Alexander Thompson, of Aurora.

For Committee of Correspondence—The present incumbents.

For Permanent Members, 1st District—Wm. B. Bibbins, Sam'l T. Hubbard, New York. 2d District—Clark A. Nicholson; Lewis H. White, of Fishkill. 3d District—J. V. Lansing, of Albany; J. R. Boulware, of Albany. 4th District—John P. Shaver, of Little Falls; Isaac J. Buckbee, of Fonda. 5th District—William

Russell, of Utica; Alonzo Churchill, of Utica. 6th District—E. G. Crafts, Binghamton; E. Odell, Unadilla. 7th District—H. D. Diadama, Salina; H. N. Eastman, Geneva. 8th District—John F. Whitbeck, of Rochester; David Little, of Rochester.

Eligible for Permanent Membership, 1st District—Edward H. Janes, New York; George F. Shrady, New York. 3d District—P. V. S. Pruyn, Kinderhook; Wm. Lamot, Charlotteville; Jacob S. Mosher, Albany; William H. Craig, Albany. 4th District—Francis Burdick; Henry H. Greene, Paine's Hollow. 5th District—J. K. Leang; Robert Frazier, Camden. 6th District—L. Griffin, Binghamton; J. W. Thompson, Schuyler Co.; J. Dolson, Bath. 7th District—A. W. Marsh, Palmyra; H. C. Hendricks, McGrawville; E. J. Schoonmaker, Magee's Corners. 8th District—M. W. Townsend, Bergen; Charles E. Rider, Rochester; Julius F. Miner, Buffalo.

For Honorary Members.—N. D. Benedict, Florida; Joseph K. Barnes, Surgeon-General U. S. A.; Isaac Ray, Providence, R. I.; Thomas S. Kirkbridge, Philadelphia.

Eligible for Election as Honorary Members.—Professor Stokes, Dublin; Prof. Rawdon McNamara, Dublin; Dr. H. C. Lombard, Geneva, Switzerland; Dr. T. R. Varick, Jersey City, N. J.; Dr. William Livingston, St. John, N. B.; Sir James Y. Simpson, Edinburgh.

For Honorary Degree of Doctor of Medicine.—Lewis Post, Lodi.

Delegates to the National Quarantine Convention.—Present incumbents.

Delegates to Connecticut State Medical Society.—J. C. Hutchison, Brooklyn; Philander Stewart, Peekskill; E. S. F. Arnold, Yonkers; Arthur S. Wolff, Plattsburgh.

Delegates to Massachusetts State Medical Society.—J. F. Jenkins, Yonkers; H. D. Bulkley, New York City; G. J. Fisher, Sing Sing; Alden March, Albany.

Delegates to New Jersey State Medical Society.—Ferris Jacobs, Delhi; Samuel Hart, Brooklyn; Frederic Hyde, Cortlandville; William Govan, Stoney Point.

Delegates to New Hampshire Medical Society.—M. R. Holbrook, Poughkeepsie; Hiram McNutt, Warrensburgh; E. R. Peaslee, New York; W. B. Bibbins, New York.

Delegates to the Vermont State Medical Society.—Drs. Francis Burdick, Johnstown; J. G. Orton, Binghamton; Thompson Burton, Fultonville.

Delegates to Pennsylvania Medical Society.—Drs. Caleb Greene, Homer; W. C. Wey, Elmira; George Burr, Binghamton; H. C. Stiles, Brooklyn.

Delegates to Ohio State Medical Society.—Drs. C. C. Wyckoff, Buffalo; Sandford Eastman, Buffalo; Thos. F. Rochester, Buffalo; H. W. Dean, Rochester; H. H. Langworthy, Rochester.

Delegates to Maine State Medical Society.—Drs. Ellsworth Eliot, New York; E. L. Beadle, Poughkeepsie.

Delegates to Rhode Island State Medical Society.—Dr. J. H. Burger, Brooklyn.

Delegates to American Medical Association.—The present incumbents, leaving out H. A. Carrington, C. S. Wood, Seth Shore, C. M. Crandall; and adding Wm. Govan, Stoney Point; Joseph Bates, James P. White, Buffalo; H. N. Eastman, Geneva; J. F. Jenkins, Yonkers; E. S. F. Arnold, Yonkers.

Committee on Statistics.—Present incumbents.

Committee on Prize Essays.—Present incumbents.

Committee on Publication and Revision of By-Laws.—Present incumbents.

Dr. C. A. Lee then proposed the following resolutions in regard to an amendment of the By-Laws to be offered for consideration at the next meeting, February, 1869:

1. *Resolved*, That the By-Laws of the New York State Medical Society, requiring the President and Vice President to be nominated by a committee appointed by the President, is hereby repealed.

2. *Resolved*, That, hereafter, in all elections for officers, the President and Vice President, it shall be done by ballot by open nomination in convention, and that whoever shall receive the largest number of votes, shall be declared elected.

3. The election shall take place on the second day of the meeting, during the morning session.

After considerable discussion, the resolutions were laid on the table, with the understanding that they be taken up and considered at the next annual meeting, the President elect also having intimated that he should call the attention of the Society to the subject, in his opening address.

Dr. J. G. Orton, through Dr. Saunders, offered the following:

*Resolved*, That the Standing Committee on Statistics be instructed to issue a suitable circular under the direction of the Publishing Committee, inviting reports from the profession of any epidemics which may prevail during the present year in this State. Adopted.

Dr. Joseph C. Hutchison offered the following:

*Resolved*, That the chairman of the delegates from this Society to the American Medical Association, be requested to present to said Association, as the desire of the Medical Society of the State of New York, the following resolution, and to urge its adoption:

*Resolved*, That the Faculties of the several Medical Colleges of the United States be recommended to announce explicitly in their annual commencement circulars and advertisements, that they will not receive certificates of time of study from irregular practitioners, and that they will not confer *the degree* upon any one who may acknowledge his intention to practice in accordance with any exclusive system. Adopted.

Business Committee read by title the following paper:

Report of Cholera at Quarantine, by Dr. John Swinburne, for 1867.

Moved that the Secretary be directed to advertise three times the prizes offered by this Society in medical journals of this State. Carried.

On motion of Dr. Bibbins, a vote of thanks to the State Agricultural Society was passed, for the gratuitous use of the Agricultural Society Hall during the session of the Society. Adopted.

Business Committee read by title Carbolic Acid, by D. P. Bissell.

Dr. Bissell moved that the thanks of this Society be tendered the Committee of Arrangements, for the good accommodations and comfortable arrangements made for this Society. Adopted.

*Resolved*, That the thanks of the members of the Medical Society of the State of New York be tendered to Drs. March and Armsby, for the kind and generous hospitality tendered to them at the Hospital Building, Wednesday evening, Feb'y 5, 1868. Adopted.

Dr. Brinsmade, on behalf of the committee appointed to memorialize the Legislature, asked that the committee be continued. Granted.

Dr. Squibb offered the following, which was adopted:

*Resolved*, That the thanks of the Society are due, and hereby tendered, to the President and other officers of the present annual session.

*Resolved*, That the thanks of the Society are tendered to the reporters who have been present, and have reported the transactions.

Dr. C. A. Lee moved that the thanks of this Society be given to Dr. E. R. Squibb, for the very able manner in which he has performed his duties as Chairman of the Business Committee. Carried.

*Does the Negro have Delirium Tremens?*—Dr. B. P. Staats stated that he had considerable experience in the treatment of delirium tremens in the County Penitentiary, and had never seen one case in a negro. He asked the experience of members present.

Dr. Brinsmade, as chairman of the committee to try the case of Dr. N. K. Freeman, reported progress, and asked that the committee be instructed to report at the next meeting. The request, after a little discussion, was granted.

The Society then adjourned to the first Tuesday in February, 1869.

